# Orthogonal translation with 5‐cyanotryptophan as an infrared probe for local structural information, electrostatics, and hydrogen bonding

**DOI:** 10.1002/pro.4705

**Published:** 2023-07-01

**Authors:** Georg Johannes von Freiherr Sass, Matthew Blain‐Hartung, Tobias Baumann, Katrina T. Forest, Peter Hildebrandt, Nediljko Budisa

**Affiliations:** ^1^ Technische Universität Berlin Institut für Chemie/Biokatalyse Berlin Germany; ^2^ Technische Universität Berlin Institut für Chemie / Physikalische Chemie Berlin Germany; ^3^ Department of Bacteriology University of Wisconsin‐Madison Madison Wisconsin USA; ^4^ Department of Chemistry University of Manitoba Winnipeg Manitoba Canada

**Keywords:** 5‐cyanotryptophan, genetic code expansion, infrared spectroscopy, local electrostatics, noninvasive probe, photoreceptor protein, vibrational stark effect

## Abstract

Orthogonal translation is an efficient tool that provides many valuable spectral probes capable of covering different parts of the electromagnetic spectrum and thus enabling parameterization of various structural and dynamic phenomena in proteins. In this context, nitrile‐containing tryptophan analogs are very useful probes to study local electrostatics and hydrogen bonding in both rigid and dynamic environments. Here, we report a semi‐rational approach to engineer a tyrosyl‐tRNA synthetase (TyrRS) variant of *Methanocaldococcus jannaschii* capable of incorporating 5‐cyanotryptophan (5CNW) via orthogonal translation. We combined one round of the well‐established positive selection system with saturation mutagenesis at preselected TyrRS positions, resulting in a novel 5CNW‐specific enzyme that also exhibits high substrate tolerance to other aromatic noncanonical amino acids. We demonstrated the utility of our orthogonal pair by inserting 5CNW into the cyanobacteriochrome Slr1393g3, a bilin‐binding photosensor of the phytochrome superfamily. The nitrile (CN) group of the inserted 5CNW provides non‐invasive labeling in the local structural context while yielding information on local electrostatics and hydrogen bonding by IR spectroscopy. 5CNW is a versatile probe that can be used for both static and dynamic measurements.

## 1 INTRODUCTION

Fluorescence of tryptophan (Trp) is a valuable intrinsic property of native proteins that can be used to study and elucidate protein structure, dynamics and function (Rubini et al., 2006; Lakowicz, 2006). The unique biophysical properties of Trp allow its involvement in numerous interactions in proteins such as π–π stacking, hydrogen bonding, cation, and van der Waals packing interactions (Budisa & Paramita Pal, 2004). Its high fluorescence quantum yield and large molar extinction coefficient for transitions to excited states enable spectral studies with highly diluted sample solutions (Khemaissa et al., 2022). Trp fluorescence, particularly its emission wavelength, serves as a crucial indicator of the measurable physicochemical characteristics within its microenvironment. These include the Stokes shift, fluorescence lifetime, and even local electrostatic fields, thereby offering valuable insights (Vivian & Callis, 2001).

However, the photophysics of Trp are often complicated because many different mechanisms are involved in shaping the excited‐state signals of the indole fluorophore in each protein environment. Despite being the least abundant amino acid residue (Barik, 2020) in proteins, most proteins contain more than one Trp and it is further notoriously difficult to parameterize local electrostatic fields without high background signals (Gosavi & Korendovych, 2016). A classical approach to study the functional role of Trp side chains in proteins is to exchange them with other aromatic canonical amino acids (tyrosine, histidine, and phenylalanine) by site‐directed mutagenesis. Despite its significance, this approach has limitations in certain cases where the strategy's effectiveness is restricted. This is particularly evident when specific Trp residues, vital for the structural and functional integrity of proteins, undergo substantial modifications in terms of size, charge or chemical nature (Budisa et al., 2004).

For this reason, chemical analogs of the canonical amino acid Trp are a reasonable alternative to enhance, modulate, and expand its spectral properties. The well‐developed synthetic chemistry and biochemistry of indoles should allow the use of non‐canonical Trp analogs and surrogates to achieve more subtle changes in side‐chain structure. Examples include the exchange of exocyclic rings at the atomic level (e.g., =CH— → =NH—; —NH— → —S—: “atomic mutations”) (Minks et al., 1999) or the addition of a functional group to the ring structure (methyl, amino, methoxy, hydroxy, nitrile groups, etc.). These discrete and usually non‐invasive changes are expected to significantly simplify the interpretation of experimental data or to introduce new biophysical properties and spectral probes.

Site‐specific incorporation of various Trp analogs by orthogonal translation was recently reported as a valid approach to alter the spectral properties of proteins (Jiang et al., 2020). By using orthogonal pairs (o‐pairs, consisting of an engineered aminoacyl‐tRNA synthetase co‐expressed with a cognate tRNA) based on *Methanosarcina mazei* pyrrolysyl‐tRNA synthetase (*Mm*PylRS), we and others were able to site‐specifically incorporate the Trp analogs 3‐benzothienyl‐L‐alanine (Bta) and 1‐methyl‐L‐tryptophan (1‐MeW) into different model proteins (Jiang et al., 2020; Tseng et al., 2020). Recently, we used *Mm*PylRS‐based and *Methanocaldococcus jannaschii* tyrosyl‐tRNA synthetase (*Mj*TyrRS)‐based o‐pairs to incorporate Trp‐like structures such as naphthyl‐alanines and β‐(1‐azulenyl)‐L‐alanine (AzAla) (Baumann et al., 2019a). We and others have also demonstrated the utility of Bta by studying the role of single hydrogen bonds in protein photophysics (Moldenhauer et al., 2023), while AzAla has proven to be an excellent molecular tool for studying the vibrational energy transfer in proteins (Deniz et al., 2021). More recently, the chimeric design of pyrrolysyl‐tRNA synthetase/tRNA pairs with phenylalanine‐tRNA identity elements enabled the ribosomal incorporation of tryptophan analogs such as 6‐cyanotryptophan and 7‐cyanotryptophan using *E. coli* and mammalian cells (Ding et al., 2020). These analogs have a much higher fluorescence quantum yield than Trp and can therefore be used for time‐resolved fluorescence measurements to study conformational features of proteins (Waegele et al., 2009).

**FIGURE 1:**
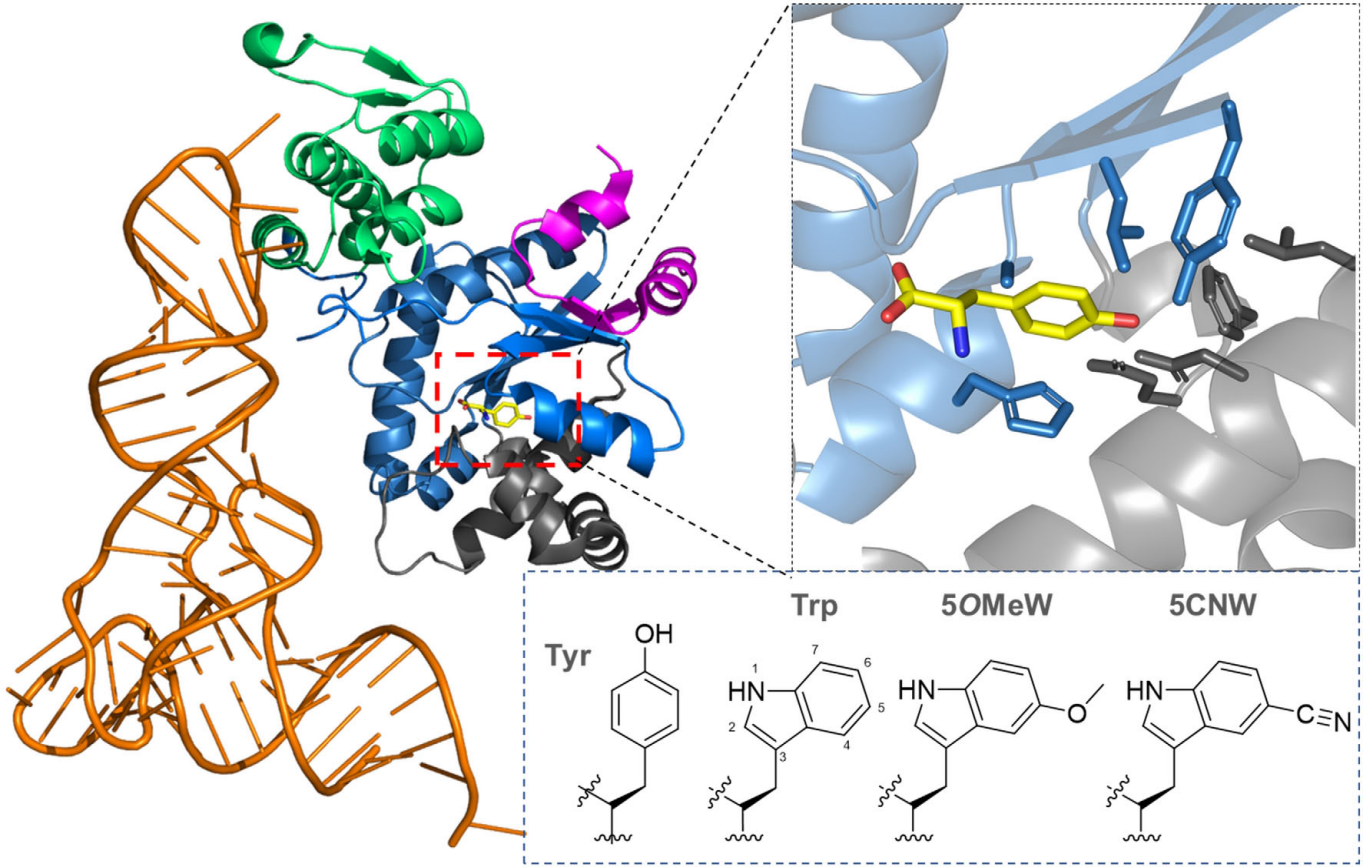
The crystal structure (PDB code: 1J1U) of monomeric *Methanocaldococcus jannaschii* (*Mj*TyrRS) in complex with its cognate tRNA^Tyr^ and its natural amino acid substrate tyrosine (Tyr). Left: Specific protein domains are highlighted as follows: magenta—N‐terminal domain; blue—Rossman fold domain; gray—CP1 domain; green —C‐terminal domain; cyan—KSMKS loop. The Tyr substrate is shown in yellow and the tRNA molecule in orange. Right: key residues surrounding Tyr within the active site pocket of *Mj*TyrRS are shown for the native structure. These were initially mutated for 5*O*MeW substrate recognition and subsequently for efficient 5CNW recognition. The natural Tyr substrate (shown in yellow) and the side chains of selected residues for saturation mutagenesis are represented as sticks. Residues ranging from Val166 to Asn193 were removed for better visibility. Insert: chemical structure of the amino acid side chains of tyrosine (Tyr), tryptophan (Trp), 5‐*O*‐Methyltryptophan (5*O*MeW) and 5‐Cyanotryptophan (5CNW). The evolved aaRS enzymes strictly discriminate Tyr and Trp but exhibit a high level of substrate tolerance (promiscuity) towards 5*O*MeW and 5CNW.

In biophysical studies, nitrile‐containing cyanotryptophans are attractive tools to manipulate the fluorescence properties of Trp through their electron‐withdrawing nitrile group. Interestingly, orthogonal pairs of 4‐cyanotryptophan and 5‐cyanotryptophan have not been previously reported. They are particularly suitable for parameterizing local interactions in proteins by IR spectroscopy due to the linear correlation between cyano groups and the electrostatics of the microenvironment (Van Wilderen et al., 2018). In this context, 5‐cyanotryptophan (5CNW) is of particular interest because it is very sensitive to local hydration in proteins (> 100‐fold compared to canonical Trp) (Markiewicz et al., 2016). This motivated us to design a novel orthogonal pair for the site‐specific incorporation of 5CNW (Figure 1), with the goal of enabling the non‐invasive labeling of target proteins with a spectroscopically valuable nitrile functionality. The applicability of the approach of non‐invasive pre‐installations of nitrile groups to address biological questions was demonstrated by performing the first biophysical studies using para‐cyanophenylalanine with myoglobin as a model protein (Schultz et al., 2006). We now have employed this approach to label the cyanobacteriochrome Slr1393g3 which is a photoreceptor protein with remarkable photochromism involving two main states (Pr and Pg) (Kraskov et al., 2021). The transformation between the Pr and the Pg states is initiated by the Z/E photoisomerization of the phycocyanobilin (PCB) chromophore (Kraskov et al., 2020). In this study, we have demonstrated the incorporation of 5CNW near the chromophore binding pocket without affecting the photoconversion. Thus, it was possible to non‐invasively monitor the changes of the electrostatics associated with the Pr/Pg transition by measuring the stretching mode of the nitrile group (Vibrational Stark Effect, VSE).

## 2 MATERIALS AND METHODS

### 2.1 One‐step positive selection–general principles

Orthogonal pairs are commonly generated by positive and negative selection cycles to identify aaRS variants dedicated to specifically charge their cognate tRNA with desired noncanonical amino acids (ncAAs), while rejecting the canonical amino acids (cAAs) (Krahn et al., 2020). However, the stringency of the negative selection cycle can lead to the removal of enzyme variants whose amino acid selectivity is sufficient in the presence of ncAA supplementation. In several instances, engineered aaRS variants were reported which allow the production of homogeneously ncAA‐labeled protein, despite leading to a background incorporation of canonical amino acids in absence of ncAA supply (Gueta et al., 2022). The utility of this subset of enzymes is obvious, but they would disappear from the aaRS pool during the negative selection cycle. In one of our previous studies (Sun et al., 2022), we performed a one‐step positive selection and omitted the negative selection stage. *E. coli* cells were co‐transformed with a plasmid encoding the chloramphenicol‐acetyltransferase (CAT) gene disrupted by an amber stop codon, the gene for the amber suppressor $\mathrm{Mj}{\text{tRNA}}_{\mathrm{CUA}}^{\mathrm{Tyr}}$ and SUMO‐His_6_‐sfGFP(R2TAG) as a reporter gene (containing a kanamycin (Kan) resistance cassette) as well as plasmids harboring an *Mj*TyrRS‐based library (with ampicillin resistance) (Hoppmann et al., 2014). The *E. coli* cells were grown in a chemically defined medium containing all canonical amino acids (cAAs), kanamycin and ampicillin (for maintaining the positive selection plasmid as well as the library plasmid) and derivatives of tryptophan as ncAAs in the presence of chloramphenicol (Cm, for positive selection pressure) (Schildhauer, 2018).

The stringency of the described positive selection can be modulated by the number of amber stop codons within the CAT gene and the concentration of the antibiotic chloramphenicol (Cm), to which functional CAT confers resistance. The amber suppression efficiency of the evolved *Mj*TyrRS enzymes can further be assessed by fluorescence assays based on read‐through of a single amber codon in‐frame of a *sf*GFP gene. Only colonies harboring functional aaRSs with adequate activity are viable (producing full‐length CAT) and show intense fluorescence (producing full‐length *sf*GFP) (Hoppmann et al., 2017). Our screening was performed in the presence of cAAs with or without the ncAA and finally coupled with aaRS gene sequencing and statistical analyses, resulting in the identification of highly efficient aaRSs.

### 2.2 One‐step positive selection for a 5*O*MeW‐specific orthogonal pair

This study was based on a *Mj*TyrRS gene library previously employed for the selection of variants charging their cognate tRNA with β‐(1‐azulenyl)‐L‐alanine (AzAla) (Baumann et al., 2019a). In this library, residues Tyr32, Leu65, Ala67, His70, Phe108, Gln109, Asp158, and Leu162 (see Figure 1) were randomized to all or a set of selected amino acids, respectively. This library was used in a positive selection round for the incorporation of 5‐*O*‐methyltryptophan (5*O*MeW) into sfGFP(R2TAG) as described elsewhere in greater detail (Schildhauer, 2018). Briefly, *E. coli* DH10b cells containing the plasmid pPAB26’ CAT(Q98TAG, D181TAG) His‐SUMO‐sfGFP(R2TAG) $\mathrm{Mj}{\text{tRNA}}_{\mathrm{CUA}}^{\mathrm{opt}}$ were transformed with a pBU18 plasmid (pUC origin of replication) encoding the *Mj*TyrRS gene library under control of the *E. coli* glnS’ promoter and terminator. Cells were incubated at 37°C for 1 h and plated out on NMM (New Minimal Media (Budisa et al., 1995) agar plates containing 1 mM 5*O*MeW, 50 μg/mL kanamycin (Kan), 70 μg/mL chloramphenicol (Cm), and 0.5 mM IPTG. After incubation at 37°C for 24 h, selected clones were harvested and tested for growth in the presence and absence of 5*O*MeW. For this purpose, clones were resuspended in ddH_2_O and spotted onto NMM agar plates spiked with 50 μg/mL Kan, 70 μg/mL Cm, 0.5 mM IPTG, and with or without the addition of 1 mM 5*O*MeW. Here, 12 out of 70 tested clones have shown selective growth only in the presence of 5OMeW.

Since 5*O*MeW and 5CNW are sterically similar substrates, we first tested these variants (sequence information is available in SI) in the whole‐cell fluorescence assay for the incorporation of 5CNW. Here, we identified a clone (5*O*MeW_RS) that showed slightly enhanced activity towards 5CNW. We further prepared it for a semi‐rational saturation mutagenesis approach to improve the catalytic performance of the aaRS enzyme (e.g., cloning of the 5*O*MeW_RS into the pULTRA vector, see below) and analyzed it in whole cell flourescence assays regarding the usability of different *E. coli* strains (see below). Here, 12 out of 70 tested clones have shown selective growth only in the presence of 5*O*MeW.

### 2.3 Plasmid backbone change of novel 5*O*MeW_RS

The 5*O*MeW_RS gene was amplified using designed primers for the integration of 5′ and 3′ overhangs with NotI restriction sites. The PCR product as well as the pULTRA vector were digested using NotI FD enzyme (Thermo Scientific (Darmstadt, Germany)), the digested fragments were purified and finally ligated using T4 DNA ligase (New England Biolabs, Frankfurt am Main, Germany). The ligation product was transformed into chemically competent *E. coli* TOP10 cells. Clones were picked and submitted for Sanger sequencing to check for correct ligation and orientation of the 5*O*MeW_RS gene.

### 2.4 Whole‐cell fluorescence assay

**FIGURE 2:**
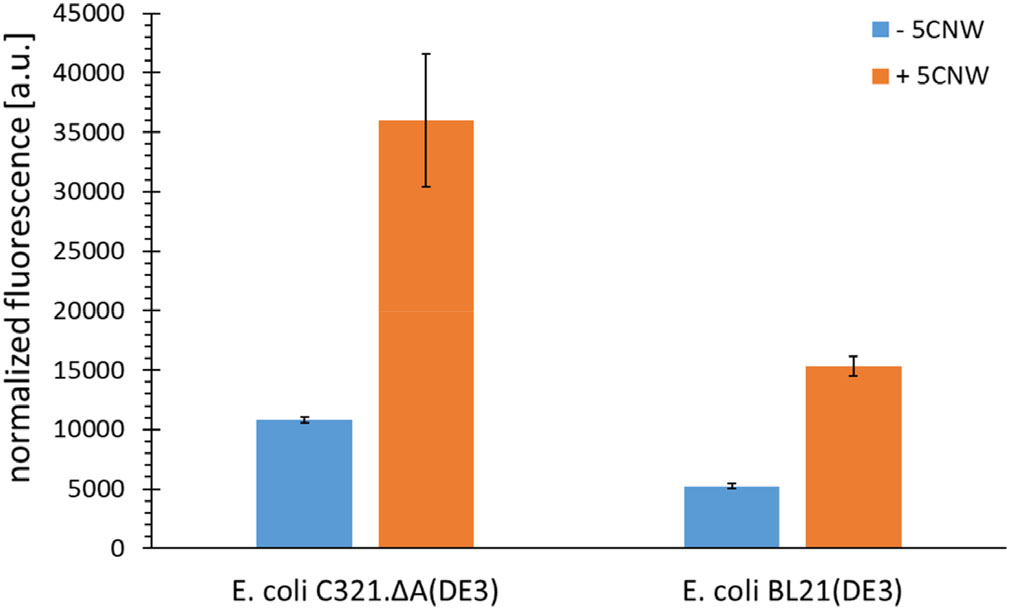
Whole‐cell fluorescence assay with 5*O*MeW_RS identified from positive selection in *Escherichia coli* BL21 (DE3) and *Escherichia coli* C321ΔA(DE3) cells. Validation and characterization of ribosomal 5CNW incorporation into SUMO‐sfGFP bearing an amber codon at the position Arg2 by 5*O*MeW_RS mutants selected for the design (Y32G, L65W, A67G, H70G, F108E, Q109S, D158A, L162D). sfGFP fluorescence assay performed with supplemented ncAA (+5CNW) or without the addition of the ncAA (‐5CNW). Fluorescence values (excitation: 481 nm, emission: 511 nm) were normalized to the lowest fluorescence value in *E. coli* C321ΔA(DE3) and to the OD_600_ of the bacterial culture. The data shown was an average of triplicate measurements (Mean ± SD).

5*O*MeW_RS obtained from the aaRS gene library originally designed for azulenyl‐alanine selection (Baumann et al., 2019a) was co‐transformed with the plasmid pET‐28 SUMO‐sfGFP(R2TAG) into chemically competent *E. coli* BL21 (DE3) or C321 (DE3) cells (Hauf et al., 2017) and spread on LB/ Spectinomycin (Spec)/Kan agar plates. Clones were collected in a sterile 96‐well microtiter plate. Each well contained 100 μL of ZYP‐5052 autoinduction medium (Studier, 2005) supplemented with 100 μg/mL Spec, 100 μg/mL Kan, and 1% (w/v) glucose. Plates were shaken overnight at 37°C and 300 rpm and covered with a gas‐permeable film (Sigma Aldrich, Taufkirchen, Germany). 1 μL of each cultivation was transferred to a fresh well of a new sterile microtiter plate containing 100 μL of ZYP‐5052 autoinduction medium supplemented with 100 μg/mL Spec, 100 μg/mL Kan, and 1 mM of the respective ncAA. The plate was incubated at 37°C and 300 rpm for 18 h. Fluorescence was determined by measuring excitation at 488 nm and emission at 530 nm using a microplate reader (Tecan, Männedorf, Switzerland) and optimal amplification (automated signal gain) with a black polystyrene microplate. In addition, the cultivations were transferred to a transparent polystyrene microtiter plate, optical density at 600 nm was measured and the fluorescence was normalized to the corresponding OD_600_ value. Biological triplicate experiments were performed for each sample. The whole‐cell fluorescence assay with 5*O*MeW_RS in BL21 (DE3) and C321 (DE3) cells is shown on Figure 2.

### 2.5 Rational search for a 5CNW‐specific enzyme—site‐directed aaRS binding pocket mutagenesis and screening of mutants

Positions Gly32, Trp65, Gly70, Glu108, Ser109, Asp162, Ala167, and Ala180 of the 5*O*MeW_RS enzyme were randomized separately using a site saturation mutagenesis approach. Primer pairs with NNK codons at the respective amino acid positions and a BsaI restriction site were used. PCR products were digested with the Type IIS restriction enzyme BsaI (Thermo Scientific, Darmstadt, Germany) and subsequently ligated with T4 ligase (Thermo Scientific, Darmstadt, Germany). Successful randomization of the different positions was verified by Sanger sequencing. 96 variants/clones were selected for each randomized position and analyzed by a whole cell fluorescence assay. The best aaRS variants were selected for sequencing, used to co‐transform *E. coli* cells with the sfGFP(R2TAG) reporter construct (see above), and analyzed in the presence and absence of 5CNW to verify maintained orthogonality. Beneficial mutations were combined via the QuikChange method and likewise analyzed. This resulted in the enzyme variant 5CNW_RS (in fact 5*O*MeW_RS_E108 W‐L109F as the best‐performing variant; vide infra), which enables orthogonal translation with 5CNW.

### 2.6 Orthogonal translation for incorporation of 5CNW into the cyanobacteriochrome Slr1393g3

*Escherichia coli* BL21 (DE3) cells were co‐transformed with plasmid pET30b_Slr1393g3_W496TAG carrying the gene for Slr1393g3 with an amber stop codon at position 496 and the second plasmid containing the orthogonal aminoacyl‐tRNA synthetase/tRNA pair (pULTRA‐5CNW_RS). ZYP‐5052 medium supplemented with 100 μg/mL Spec and 100 μg/mL Kan was inoculated with a fresh overnight culture at a ratio of 1:100. Cells were grown under vigorous shaking (200 rpm, 37°C) until they reached the mid‐log phase of *E. coli* growth (OD_600_ value of 0.6–0.8). 5CNW was added to the cultivation at a final concentration of 1 mM and the cell suspension was cooled to the desired temperature. Protein expression took place for 18 h (150–200 rpm, 20°C). Cells were harvested by centrifugation (5000 × *g*, 4°C, 20 min) and stored at −80°C until further processing.

### 2.7 Cell disruption and protein purification

*Escherichia coli* cell pellets were resuspended in lysis buffer (50 mM Tris, 500 mM NaCl, 0.1 mM PMSF), 20 μg/mL DNase, 20 μg/mL RNase and 50 μg/mL lysozyme were added, and the cell suspension was incubated on ice for 1 h. Cells were disrupted using a M‐110 L Microfluidizer processor (Microfluidics, Westwood, MA), cell debris was removed by centrifugation (15,000 × *g*, 4°C, 60 min) and the supernatant was filtered (0.45 μm) to recover the cell lysate.

Protein purification took place on an ÄKTA FPLC system using 1 or 5 mL HisTrap FF crude columns (GE Healthcare, München, Germany). The system and the column were equilibrated with washing buffer (50 mM Tris, 500 mM NaCl, pH 8.0 at 4°C) prior application of the cell lysate. The column was subsequently washed with 20–30 column volumes (CV). Next, the target protein was eluted from the Ni‐NTA column by applying an imidazole gradient ranging from 20 to 500 mM (using the elution buffer containing 50 mM Tris, 300 mM NaCl, 500 mM Imidazol, pH 8.0 at 4°C). Protein containing fractions were pooled and rebuffered in storage buffer (20 mM Tris, 150 mM NaCl, 0.1 mM PMSF, 20% glycerol, pH 8.0 at 4°C) and the protein concentration was determined via Bradford assay.

### 2.8 Electrospray ionization mass spectrometry with liquid chromatography (LC‐ESI‐QTOF‐MS)

The molecular mass of purified intact proteins was determined by High‐Performance Liquid Chromatography (HPLC) coupled to Electrospray Ionization Time‐of‐flight Mass Spectrometry (LC‐ESI‐TOF‐MS). Protein samples were injected at a concentration of 0.1–0.2 mg/mL and a flow rate of 0.3 to 0.5 mL/min. Reversed‐phase chromatography was performed by applying a 20 min linear gradient from 5% to 80% acetonitrile (in 0.1% formic acid in ddH_2_O), through a C5 column (Supelco analytical, Sigma‐Aldrich, St. Lois). Obtained spectra were deconvoluted using the maximum entropy deconvolution algorithm by the software Agilent MassHunter Qualitative Analysis (version B.06.00).

### 2.9 Spectroscopic experiments

UV–vis absorption and IR spectroscopic measurements were carried out as described in detail elsewhere (Kraskov et al., 2020).

## 3 RESULTS

### 3.1 Performance of 5OMeW_RS identified from one‐step positive selection

In this study, we reused a pre‐existing aaRS gene library initially designed for the activation of azulenyl‐alanine (Baumann et al., 2019a). Generated via Golden Gate cloning, a set of eight amino acid residues in the binding pocket of *Mj*TyrRS had been randomized to either all or to restricted sets of amino acids, respectively (i.e., semi‐rational approach). Derived from this library, a panel of selected engineered *Mj*TyrRS mutants showed improved performance towards activation of tryptophan analogs, leading to their ribosomal incorporation into the model fluorescent protein sfGFP (Schildhauer, 2018). We were particularly interested in the mutant 5*O*MeW_RS (Y32G, L65W, A67G, H70G, F108E, Q109S, D158A, L162D; Figure 1) which is capable to activate 5*O*MeW. It was used as starting point to search for an aaRS enzyme capable of recognizing a similar substrate, 5CNW.

In initial orthogonal translation tests, we used either *E. coli* BL21 (DE3) or C321ΔA(DE3) cells for quantifying the ncAA incorporation efficiency obtained via the 5OMeW_RS variant. In both cell lines, an increased fluorescence by a factor of ca. three in the presence of 5CNW as well as preserved orthogonality (no significant reporter protein production) in its absence was detected. As the background suppression in *E. coli* C321ΔA(DE3) cells was significantly higher than in BL21 (DE3) cells, the latter strain was used for further investigations. Due to the detectable (promiscuous) activity of 5OMeW_RS towards 5CNW, this *Mj*TyrRS variant presented a good starting point for further enzyme engineering.

### 3.2 Randomization of single residues in the 5*O*MeW_RS binding pocket

Saturation mutagenesis in the binding pocket of the *Mj*TyrRS variant led to different fluorescence reporter results depending on the position of the mutation (see Figure S1–S9). The most promising variants with preserved orthogonality were sequenced and analyzed with the following results: mutations at position 32 did not increase the fluorescence signal compared to 5*O*MeW_RS, indicating that Gly at this position is already the best choice. Interestingly, mutations at position 65 resulted in a significantly decreased signal in almost all cases, indicating that Trp at this position is essential for 5CNW detection.

Moreover, it clearly shows that this aaRS position does not play a role in the orthogonality of 5*O*MeW_RS. The same is true for residues 162 and 180, where mutations also led to a drastic loss of enzyme activity in almost all variants. Randomization of position 70 resulted in a high number of increased fluorescence signals. However, the background activities observed revealed that this was due to loss of orthogonality, suggesting that Gly is a gatekeeper residue for orthogonality (see Figure S10). Mutations at position 167 resulted in a drastic loss of activity in most cases and a slight increase in fluorescence signal in a few cases. However, subsequent orthogonality tests showed that the latter was again only due to increased background suppression. Gratifyingly, an improvement in ncAA incorporation efficiency was achieved by mutations at positions 108 and 109. Here, variant 108 W as well as variants 109A and 109 L showed an overall improvement with up to a 2‐fold increase in reporter signal (109 L). Mutations 109F and 109G resulted in activities comparable to the original 5*O*MeW_RS enzyme. Moreover, several mutations at these positions led to a significant reduction in background suppression, which was particularly true for mutations 108 W and 109 L. When the 108 W mutation was combined with 109 L or 109F, the aaRS efficiency was even slightly further improved (see Table 1).

**TABLE 1:** Reporter fluorescence intensities upon co‐expression of 5*O*MeW_RS as well as selected single and double mutants.

*Mj*TyrRS variant	+5CNW			–5CNW			+ 5CNW/–5CNW		
5*O*MeW_RS	100.00	±	0.91	32.68	±	0.21	3.06	±	0.05
108 W	21.01	±	0.79	5.25	±	0.27	4.00	±	0.36
109A	37.14	±	0.32	9.76	±	0.34	3.81	±	0.17
109F	70.80	±	2.45	26.90	±	0.90	2.63	±	0.18
109 L	27.04	±	0.05	4.46	±	0.04	6.07	±	0.07
109G	37.18	±	0.37	12.98	±	1.57	2.86	±	0.37
108 W‐109G	24.43	±	0.25	5.19	±	0.11	4.71	±	0.15
108 W‐109A	22.51	±	0.35	4.07	±	0.03	5.53	±	0.13
108 W‐109 L	38.01	±	4.96	6.22	±	0.16	6.12	±	0.96
108 W‐109F	61.57	±	0.82	9.94	±	0.11	6.19	±	0.15

*Note*: A whole‐cell fluorescence assay was performed with supplemented ncAA (+5CNW) or without the addition of ncAA (‐5CNW). The quotient of both values (last column) indicates the ncAA incorporation efficiency. The assay was performed in biological triplicates and the standard deviation is shown. The fluorescence intensity resulting from the original 5*O*MeW_RS setup cultured in the presence of 5CNW was normalized to 100%.

### 3.3 Substrate specificity of the novel 5CNW_RS enzyme

**FIGURE 3:**
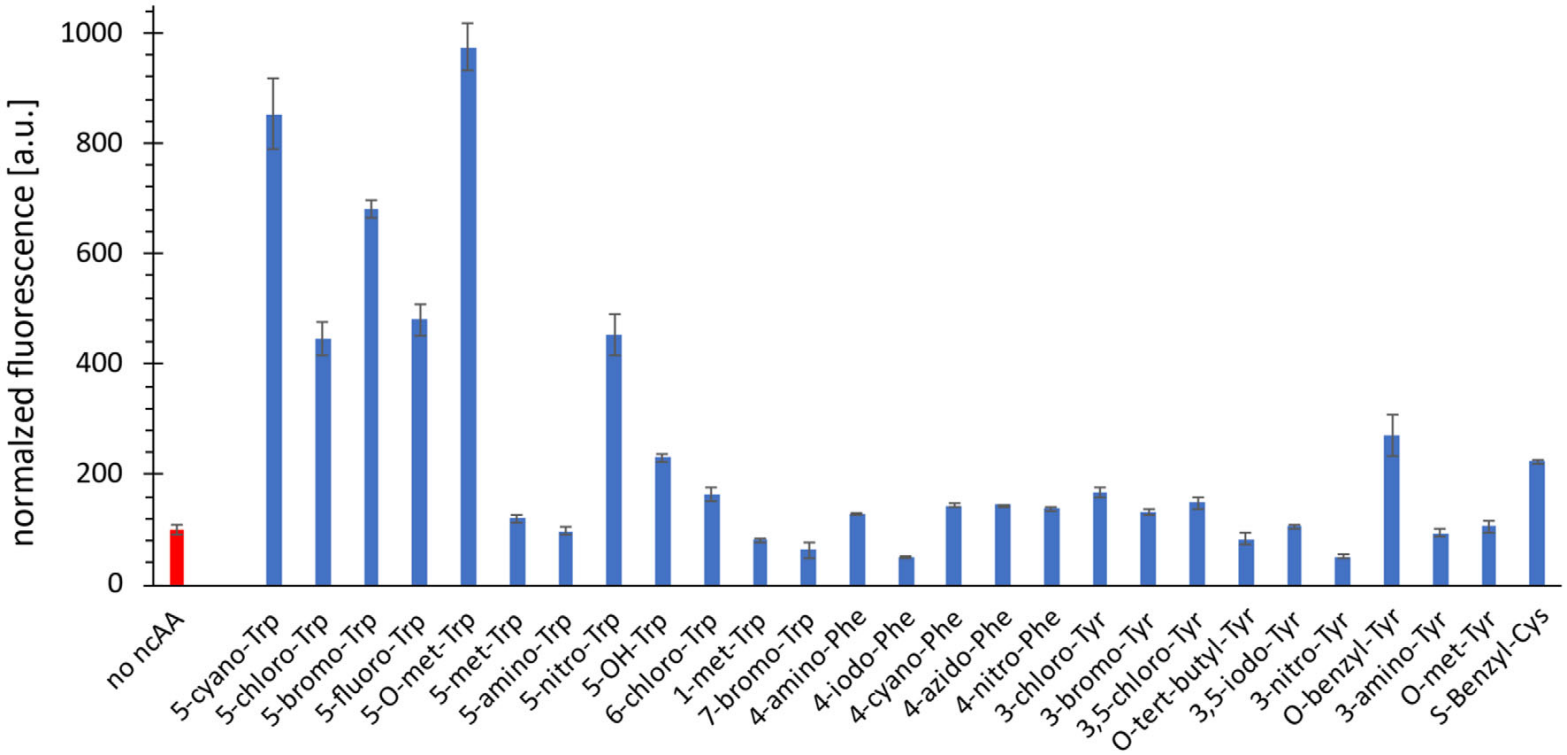
Whole‐cell fluorescence assay with 5CNW_RS using different ncAAs. The obtained fluorescence of the cultivation without addition of any ncAA was set to 100%. The assay was performed in biological triplicates and the standard deviation is shown as error bars.

The best performing aaRS variant was 5*O*MeW_RS‐E108 W‐S109F (referred to as 5CNW_RS in this study). This enzyme was evaluated in whole‐cell fluorescence assays for its activity towards ribosomal incorporation of other, mainly aromatic ncAAs (Figure 3). Reporter signals observed upon supplementation of 5‐methoxy‐Trp (5*O*MeW) were even higher compared to those of 5CNW. This was not surprising given the chemical and sterical similarity of the two ncAAs. In this way, we further improved the starting enzyme 5*O*MeW_RS towards activation and/or *Mj*tRNA charging with 5*O*MeW. Interestingly, 5CNW_RS further showed significant activity towards halogenated Trp analogs (5‐fluoro‐, 5‐chloro‐, 6‐chloro‐, and 5‐bromotryptophan) as well as for a nitro‐substituted tryptophan (5‐nitro‐Trp). We also found that the ribosomal incorporation signals of 5‐hydroxytryptophan (5‐OH‐Trp), *O*‐benzyl‐Tyr, and *O*‐benzyl‐Cys were significantly higher than control values.

### 3.4 In vivo labeling of Slr1393g3 with 5CNW

**FIGURE 4:**
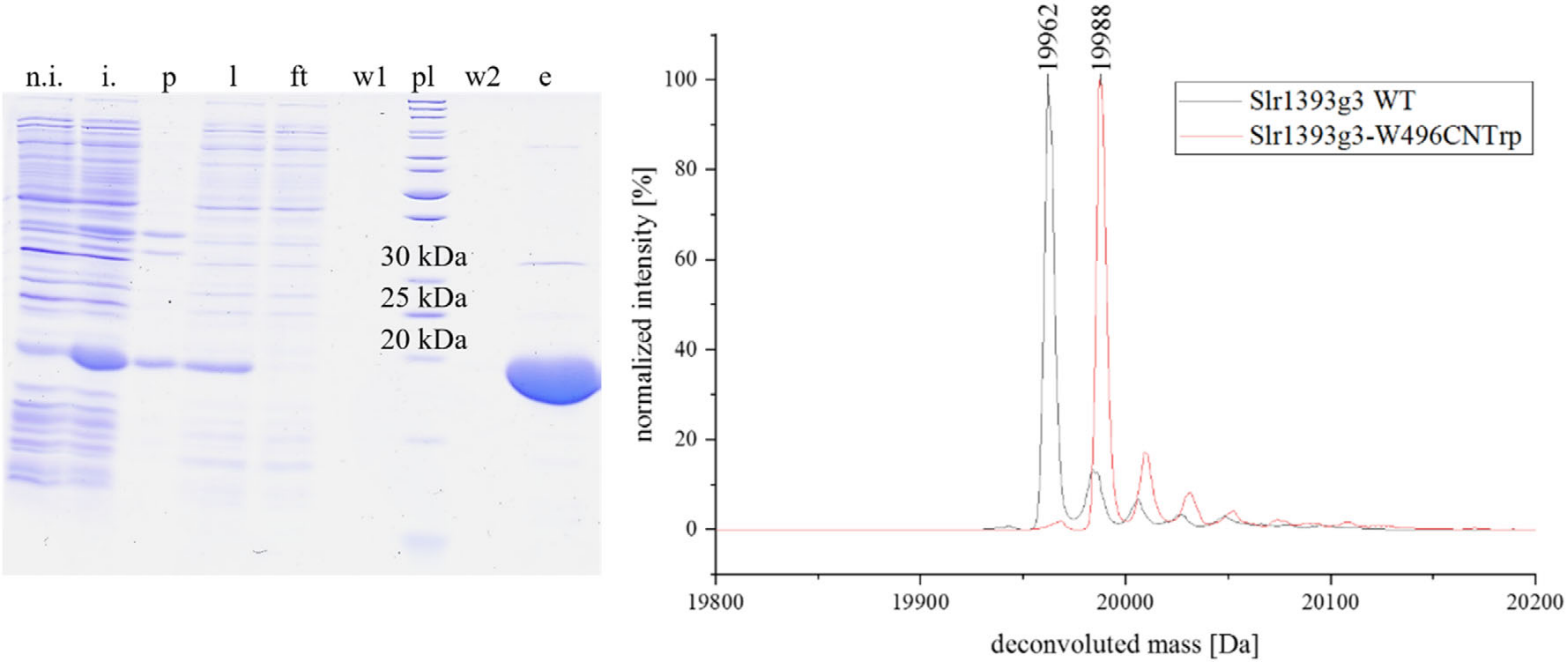
Profiles of SDS‐PAGE and HPLC‐ESI‐QTOF‐MS analysis of Slr1393g3‐W496/5CNW expression and purification. Left: SDS‐PAGE analysis shows overexpression of the target protein and sufficient purity of the sample after Ni‐NTA purification. Lanes and samples: n.i, non‐induced cultivation sample; i, cultivation sample after induced gene expression; p, pellet/insoluble fraction after cell disruption; l, lysate after cell disruption; ft, flow‐through of the Ni‐NTA purification; w1, first wash; w2, second wash; e, eluate of Ni‐NTA purification; pl, protein ladder. Right panel: deconvoluted mass (ESI‐MS) of Slr1393g3‐W496/5CNW matches the expected and calculated intact protein mass of 19,988 Da. The spectrum reveals no other peaks attributable to background suppression impurities (e.g., ribosomal Trp incorporation at Slr1393g3 position 496). Analysis of the wild‐type protein gave the calculated molecular mass of 19,962 Da.

**FIGURE 5:**
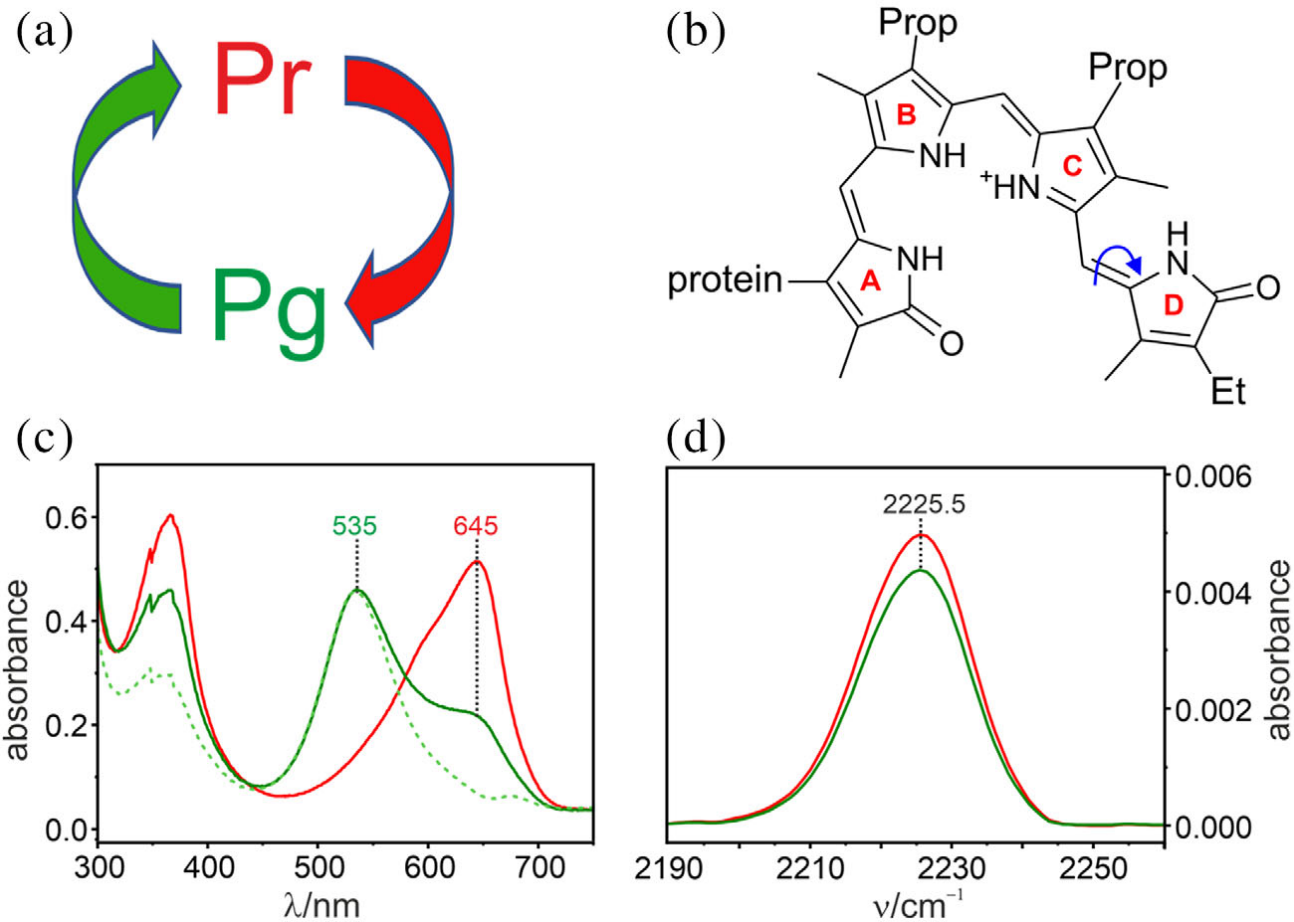
Spectral properties and photoconversion of Slr1393g3. (a) simplified schematic presentation of the Slr1393 photoswitch with the red and green arrows indicating the photoinduced reactions between Pr and Pg states. (b) structural formula of the phycocyanobilin chromophore in the ZZZssa configuration (Pr); the curved arrow indicates the isomerization to the ZZEssa configuration upon photoexcitation. (c) absorption spectra of the Pr state (red) and the photoproduct of the Slr1393g3‐W496CNW variant obtained upon red‐excitation which is dominated by the Pg state (green); the green dashed line indicates the spectrum of the pure Pg state after subtracting the residual contribution of Pr. (d) IR spectra of the Slr1393g3‐W496CNW variant in the nitrile stretching region. The red and green lines refer to the Pr and Pg state, respectively.

The novel 5CNW_RS was used to label the GAF3 domain of the cyanobacteriochrome Slr1393 (Slr1393g3) with 5CNW at the critical position 496. Significant overexpression of the target gene and purification by Ni^2+^‐based affinity chromatography were achieved. An efficient stop codon suppression could be demonstrated with an overall yield of 33 mg labeled Slr1393g3 per liter of cultivation. Using Sumo‐*sf*GFP as target protein, a yield of 75 mg labeled Sumo‐*sf*GFP per liter of cultivation was obtained (see Figure S10), which is in great accordance with the efficiency of other *Mj*TyrRS‐based orthogonal pairs (Baumann et al., 2019a). Successful protein labeling was detected by a + 26 Da mass shift in LC‐ESI‐MS measurements (Figure 4 and Figure S11). Slr1393 is a biological photoswitch that can be interconverted by light between the parent states Pr and Pg, respectively. These processes are initiated by the photoisomerization of the phycocyanobilin chromophore (Figure 5a, b). The Pr (red‐absorbing) and Pg (green‐absorbing) states, in which the chromophore adopts a ZZZssa and ZZEssa configuration, respectively, can readily be distinguished based on their UV–Vis absorption spectra (Xu et al., 2020). In the wild‐type protein, the lowest‐energy electronic transitions are found at 649 nm (Pr) and 536 nm (Pg) which are close to those of the W496CNW variant (Figure 5c). Also, photoswitching is preserved in the non‐natural protein (Figure 5d).

These findings indicate that the W → 5CNW substitution does not affect the structure or photoinduced reactions of the Slr1393g3 chromophore. Hence, the ncAA modification can be considered as non‐invasive for the photoreceptor. We were further able to detect the nitrile stretching mode of 5CNW by IR spectroscopy (Figure 5d). Its frequency in Pr at 2225.2 cm^−1^ is consistent with a nitrile group in a polar environment (Van Wilderen et al., 2018). The frequency only slightly changes upon transition to the Pg state, implying that the nitrile function does not experience a significant change of the local electrostatics despite the re‐orientation of the indole ring associated with the Pr → Pg transition (Xu et al., 2020).

## 4 DISCUSSION AND OUTLOOK

### 4.1 Development of a novel orthogonal pair for ribosomal 5CNW incorporation

Ideally, reprogramming protein translation with non‐canonical amino acids (ncAAs) requires (a) their metabolic compatibility and tolerance, (b) translational orthogonality of the artificial aaRS:tRNA pair, and (c) high fidelity ribosomal synthesis in an intact living cell or organism (Agostini et al., 2017). The orthogonality in this context means that directed evolution of aminoacyl‐tRNA synthetases (aaRSs) should yield robust enzyme variants capable of selectively recognizing their cognate tRNA and ncAAs in the pool of endogenous tRNAs, aaRSs, metabolic intermediates (e.g., homoserine, homocysteine, citrulline, ornithine, etc.) and 20 canonical amino acids (Koch et al., 2022). In this study, we were able to generate an orthogonal 5CNW_RS variant that meets these criteria and has high activity towards 5CNW, which is also a substate for cellular uptake systems and metabolically well tolerated in the *E. coli* cytosol. 5CNW_RS has high specificity for 5CNW compared to Trp but exhibited substrate promiscuity towards many unique ncAAs like 5CNW.

Our starting sequence was 5*O*MeW_RS, encoding an *Mj*TyrRS variant. This enzyme was generated by using a simplified one‐round positive selection approach (i.e., without a negative selection step, cf. Hoppmann et al., 2014) and an aaRS library randomized at eight positions. Positive selection and aaRS activity screening were conducted using genes of an antibiotic resistance marker (chloramphenicol acetyltransferase with two in‐frame amber stop codons) and a superfolder green fluorescent protein (*sf*GFP, with one in‐frame amber stop codon) reporter construct, respectively. Compared to the traditional aaRS evolution approach which employs iterative cycles of positive and negative selections, one positive selection round with activity readout was sufficient to obtain an aaRS variant with improved activity. As evident from the observed low background activities, a negative selection cycle could be omitted since a sufficient level of aaRS orthogonality was maintained. This method has been used frequently and has proven to be an efficient aaRS selection method (Hoppmann et al., 2017). Having confirmed 5*O*MeW_RS as a suitable starting point for the semi‐rational design of 5CNW_RS, we identified eight different mutation sites in the modeled amino acid substrate binding pocket of the enzyme. Typically, *Mj*TyrRS residues Tyr32 and Asp158 are replaced in engineered variants because they form hydrogen bonds with the hydroxy function of the natural substrate tyrosine. Therefore, it is believed that mutations at these sites are required to maintain orthogonality in newly constructed variants (Baumann et al., 2019b). Substitutions A67G and D158A further enlarge the binding pocket and allow binding of larger ncAAs such as 5CNW (see Figure 2). Finally, mutation of Leu65 to the aromatic amino acid Trp may be important for both π–π interactions and binding mediation of 5CNW, as previously described for an *Mj*TyrRS variant for Azulenylalanine (AzAlaRS) (Baumann et al., 2019a). The overview of the residual differences between native MjTyrRS, AzAlaRS, 5*O*MeW_RS, and 5CNW_RS enzymes and simple models are shown in Figures S11 and S12.

The overall performance of 5*O*MeW_RS for genetic encoding of 5CNW was rather subpar compared to other orthogonal *Mj*TyrRS pairs (Schildhauer, 2018), so further improvement was sought by randomizing individual residues. Saturation mutagenesis was performed to explore the full side chain repertoire. This resulted in the identification of an enzyme variant that doubles the 5CNW incorporation efficiency and greatly reduced background suppression. In addition, randomization of selected residues provided insights into the importance of individual residues for ncAA recognition or orthogonality preservation, respectively. While residues Trp65, Asp162 and Ala180 were essential for 5CNW recognition, Gly70 was found to be a gatekeeper residue for orthogonality of the aaRS backbone. Strikingly, the importance of the His70Gly mutation for increasing the active site of the synthetase has already been highlighted for AzAla (Baumann et al., 2019a). The only positions where fine‐tuning of the incorporation efficiency seemed to be possible were 108 and 109, where replacements led to a significant reduction in background suppression and to a significant increase in incorporation efficiency. It is possible that the E108 W mutation introduces another π‐π interaction with Trp analogs, thereby suppressing nonspecific binding of other amino acid substrates. At position 109, the introduction of small aliphatic residues such as Leu, Gly, or Ala resulted in a significant decrease in background suppression. However, the L109F mutation in combination with the E108 W mutation showed the best synergistic effect and resulted in the most powerful variant for ribosomal 5CNW incorporation, 5*O*MeW_RS_E108 W‐L109F (5CNW_RS). Since increased 5CNW incorporation is found to be paired with a low background suppression, we can assume that the aaRS improvements stem from an overall improved ncAA substrate recognition and binding by the enzyme. From a structural point of view, it is fitting that the two consecutive aromatic residues enhance the aaRS activity towards 5CNW (see also Figure S11 and Figure S12).

### 4.2 5CNW incorporation as a valuable spectral probe for protein biophysics

We hypothesized that an aaRS enzyme specific for 5CNW could serve as an excellent tool for introducing spectroscopically valuable nitrile group modifications via orthogonal translation. Accordingly, 5CNW may complement *para*‐cyanophenylalanine (pCNF) as a reporter group for the VSE (Biava et al., 2018; Voeller et al., 2017). Slr1393g3 was an ideal protein target to explore the potential of this label since, first, a possible impact of the substitution on structure and functionality of its photoactive site could readily be assessed by UV–Vis absorption spectroscopy. Here we could in fact demonstrate that such in vivo labeling was non‐invasive. Second, this photoreceptor includes a Trp residue that undergoes an orientational change during the Pr/Pg photoconversion (Kraskov et al., 2020). Therefore, its replacement by 5CNW introduced a molecular probe at a mechanistically important site of the protein. The corresponding nitrile stretching signal could be detected successfully. No significant change could be observed for the Pr → Pg transition. A detailed analysis of the underlying electrostatic changes will be presented elsewhere. In summary, the present results show that the site‐specific and non‐invasive substitution of 5CNW provides an important tool for probing local electrostatics and hydrogen bonding interactions.

From an engineering perspective, our approach clearly demonstrates the benefits of a semi‐rational approach involving only a small number of aaRS residues, and even randomizing individual residues. The combined strategy leads to a good exploration of the available aaRS sequence space. The resulting ncAA‐specific activity and background suppression levels observed by reporter production allow a detailed investigation of the function and significance of the different residues and combinations thereof in the active site of the synthetase. Once an orthogonal pair with significant activity for incorporation of the desired ncAA is found, randomization of individual residues provides a suitable method to further improve the enzyme and fine‐tune its scope for numerous substrates with large chemical side‐chain variability. In this study, the combination of the one‐round positive selection system with site saturation mutagenesis at selected positions led to a novel *Mj*TyrRS variant. This enzyme variant allows incorporation of the valuable spectroscopic probe 5CNW and additionally exhibits a high substrate promiscuity to other aromatic ncAAs.

### 4.3 Attributes and perspectives of the newly designed 5CNW_RS

In vivo incorporation of Trp analogs such as azatryptophans, fluorinated and hydroxylated tryptophans was reported more than five decades ago, while at the beginning of this century methylated and amine analogs as well as thienopyrrole surrogates became known as tools for engineering structural and functional properties of proteins (Budisa, 2004). Many of these noncanonical analogs of Trp have been incorporated mainly in auxotrophic bacterial cells such as *E. coli*. Such bacterial cells are generally capable of taking up such amino acids, activating and replacing to a high degree Trp positions in recombinantly expressed proteins with related analogues (Agostini et al., 2020). With plasmid‐directed recombinant protein expression and optimized fermentation, it is possible to achieve high level of residue specific substitutions (Budisa et al., 1999). This phenomenon is due to the inability of aminoacyl‐tRNA synthetases, crucial enzymes in genetic code interpretation, to discriminate between substrates that are structurally and chemically similar known as a substrate tolerance or substrate promiscuity (Hoesl & Budisa, 2012). Although this method has advantages, such as labeling in complicated enzyme‐cofactor systems (Völler et al., 2017) and especially for replacing multiple residues (Hoesl et al., 2011) with an appropriate analog, it is generally less suitable for individual site‐specific labeling.

The tolerance to different amino acid substrates and the mutational plasticity of the first and second shells of the catalytic residues is astonishing in the light of the high specificity and accuracy of aminoacyl‐tRNA synthetases (Hauf et al., 2017). Notably, TyrRS enzymes do not exhibit editing (Brick et al., 1989), which has been recognized as an advantage for active site engineering to gain new substrate specificities (Kobayashi et al., 2003). The eubacterial and yeast enzymes even load cognate tRNA^Tyr^ with D‐tyrosine and for correction of this “error,” the resulting D‐Tyr‐tRNA^Tyr^ is hydrolyzed by a specific deacylase (in the absence of this enzyme, some D‐amino acids are toxic to cells) (Sheoran & First, 2008). A sufficiently high thermostability of the enzyme also benefits mutation of the substrate binding pocket by buffering the commonly accompanying negative effects on solubility and function in bacterial hosts. For example, we (Baumann et al., 2019b) and others (Armen et al., 2010) have shown that the engineered aaRS mutants generally exhibit significantly lower thermostability compared with their wild‐type counterparts. It is thus not surprising that, for example, *E. coli* TyrRS derived from a mesophilic bacterium may represent a less stable scaffold with a lower tolerance to active site mutations (Grasso et al., 2021). Other contemporary studies have further confirmed that thermostable enzymes would be the best choice for engineering and utilizing orthogonal systems for the incorporation of noncanonical amino acids (Wals & Ovaa, 2014; Drienovská & Roelfes, 2020).

In this context, our newly designed 5CNW_RS showed a broad substrate tolerance and thus can be considered as an enzyme designed for C5‐substituted Trp analogs without the ability to finely discriminate between chemically and sterically similar substituents. Remarkably, the incorporation efficiency of 5‐*O*MeW, an antimetabolite traditionally used to study tryptophan metabolism and its role in protein biosynthesis (Barlati & Ciferri, 1970) was even higher than that of 5CNW. This was not too surprising as the initial aaRS variant 5‐*O*MeW_RS was selected for 5‐*O*MeW incorporation. The derived 5CNW_RS essentially contains mutations for a lowered background suppression, while still allowing for the recognition of chemically and sterically similar Trp analogs. Moreover, 5CNW_RS allows the genetic encoding of halogenated Trp derivatives, and thus can serve as an excellent tool to study different Trp modifications or to introduce spectroscopic probes, such as selective fluorination for ^19^F NMR. It should be noted that some chemically different analogues such as *O*‐benzyl‐tyrosine and *S*‐benzyl‐cysteine are also recognized by 5CNW_RS. Our findings presented in Figure 3 qualifies 5CNW_RS as the first *Mj*TyrRS‐derived orthogonal pair for the incorporation of 5‐nitrotryptophan with sufficiently high activity towards 5‐hydroxytryptophan incorporation. 5‐nitro‐Trp may be of particular interest and the new 5CNW_RS can usefully complement the existing orthogonal pairs for nitro‐containing ncAAs. The site‐specific incorporation of these ncAAs displays a useful tool for the investigation of diseases related to reactive nitrogen species (RNS) (Koch et al., 2022). In summary, we succeeded to generate an orthogonal enzyme with a remarkable amino acid substrate tolerance, an aaRS feature known as substrate promiscuity or even “poly‐specificity.” (Guo et al., 2014).

On first sight, the activation and tRNA charging of Trp analogs by a TyrRS enzyme may appear surprising. However, the strong similarity and high homology between the amino acid binding domains of tryptophanyl‐tRNA synthetase (TrpRS) and TyrRS from *Geobacillus stearothermophilus* has been a well‐known phenomenon for decades. Detailed mechanistic analyses of amino acid recognition and aminoacylation provided evidence for a common origin (Doublié et al., 1995). The sequence similarity of structurally superimposable residues in TrpRS and TyrRS suggests that they diverged more recently than most aminoacyl‐tRNA synthetases. In this context, TrpRS is thought to have a relatively short evolutionary history, as Trp is considered to be the most recent addition to the genetic code (Kubyshkin & Budisa, 2019). Also phylogenetic analyses of ancestral sequence reconstructions using TrpRS and TyrRS as paralogous protein families have shown that Trp is the most recent addition to the standard amino acid repertoire of the genetic code through aaRS divergence and neofunctionalization (Fournier & Alm, 2015).

The evolutionary scenario of aaRS‐mediated incorporation of Trp into the genetic code as the final step in the evolution of the genetic code to its current form should be an excellent basis for repurposing *Mj*TyrRS for the recognition of Trp analogs. We believe that the smaller phylogenetic distance between these two aaRS systems is a good indicator of the feasibility of the repurposing attempts. Orthogonal pairs destined for Trp analogs such as 5‐hydroxytryptophan (Zhang et al., 2004) have long been known, but until recently (Kwok et al., 2019) there has been no systematic attempt to incorporate large numbers of Trp analogs using in‐frame stop codon suppression methods.

## AUTHOR CONTRIBUTIONS

**Georg Johannes Freiherr von Sass:** Data curation (equal); formal analysis (equal); investigation (equal); methodology (equal); validation (equal); visualization (equal); writing – original draft (equal); writing – review and editing (equal). **Blain‐Hartung Matthew:** Formal analysis (equal); investigation (equal); methodology (equal); validation (equal). **Tobias Baumann:** Conceptualization (equal); data curation (equal); formal analysis (equal); investigation (equal); methodology (equal); software (equal); supervision (equal); validation (equal); visualization (equal); writing – review and editing (equal). **Katrina T. Forest:** Data curation (equal); formal analysis (equal); funding acquisition (equal); investigation (equal); methodology (equal); software (equal); supervision (equal); validation (equal); visualization (equal); writing – review and editing (equal). **Peter Hildebrandt:** Conceptualization (equal); data curation (equal); formal analysis (equal); funding acquisition (equal); investigation (equal); methodology (equal); project administration (equal); supervision (equal); validation (equal); visualization (equal); writing – review and editing (equal). **Nediljko Budisa:** Conceptualization (equal); data curation (equal); formal analysis (equal); funding acquisition (equal); investigation (equal); methodology (equal); project administration (equal); supervision (equal); validation (equal); visualization (equal); writing – original draft (equal); writing – review and editing (equal).

## CONFLICT OF INTEREST STATEMENT

The authors declare no conflicts of interest.

## Supporting information


**Figure S1:** Whole‐cell fluorescence assay with 5CNW_RS variants randomized at position 32. 96 single colonies were assayed in the presence 5CNW and sfGFP expression was monitored via fluorescence measurements. The bar on the far‐right side displays the fluorescence yielded with 5CNW_RS (WT).
**Figure S2:** Whole‐cell fluorescence assay with 5CNW_RS variants randomized at position 65. 96 single colonies were assayed in the presence 5CNW and sfGFP expression was monitored via fluorescence measurements. The bar on the far‐right side displays the fluorescence yielded with 5CNW_RS (WT).
**Figure S3:** Whole‐cell fluorescence assay with 5CNW_RS variants randomized at position 70. 96 Single colonies were assayed in the presence 5CNW and sfGFP expression was monitored via fluorescence measurements. The bar on the far‐right side displays the fluorescence yielded with 5CNW_RS (WT).
**Figure S4:** Whole‐cell fluorescence assay with 5CNW_RS variants randomized at position 108. 96 single colonies were assayed in the presence 5CNW and sfGFP expression was monitored via fluorescence measurements. The bar on the far‐right side displays the fluorescence yielded with 5CNW_RS (WT).
**Figure S5:** Whole‐cell fluorescence assay with 5CNW_RS variants randomized at position 109. 96 single colonies were assayed in the presence 5CNW and sfGFP expression was monitored via fluorescence measurements. The bar on the far‐right side displays the fluorescence yielded with 5CNW_RS (WT).
**Figure S6:** Whole‐cell fluorescence assay with 5CNW_RS variants randomized at position 162. 96 single colonies were assayed in the presence 5CNW and sfGFP expression was monitored via fluorescence measurements. The bar on the far‐right side displays the fluorescence yielded with 5CNW_RS (WT).
**Figure S7:** Whole‐cell fluorescence assay with 5CNW_RS variants randomized at position 167. 96 single colonies were assayed in the presence 5CNW and sfGFP expression was monitored via fluorescence measurements. The bar on the far‐right side displays the fluorescence yielded with 5CNW_RS (WT).
**Figure S8:** Whole‐cell fluorescence assay with 5CNW_RS variants randomized at position 180. 96 single colonies were assayed in the presence 5CNW and sfGFP expression was monitored via fluorescence measurements. The bar on the far‐right side displays the fluorescence yielded with 5CNW_RS (WT).
**Figure S9:** Whole‐cell fluorescence assay with selected 5CNW_RS variants tested for ribosomal incorporation of 5CNW. The assay was performed with supplemented ncAA (+5CNW) or without its addition (‐5CNW). It took place in biological triplicates and the standard deviation is depicted as error bars. The fluorescence obtained by expression of the initial 5OMeW_RS (WT) enzyme in presence of 5CNW was normalized to 100%.
**Figure S10:** HPLC‐ESI‐QTOF‐MS analysis of 5CNW incorporation into sfGFP. Deconvoluted mass of purified SUMO‐sfGFP WT and SUMO‐sfGFP proteins with 5CNW incorporated at position 2. The measured and expected molecular masses are as follows: SUMO‐sfGFP WT: measured: 40208 Da, expected: 40208.15 Da. Sumo‐sfGFP_R2(5CNW): measured: 40263 Da, expected: 40263.17 Da. The secondary peaks (40,229 Da and 40,284 Da, respectively) represent sfGFP derivatives with immature chromophore, as evident from the wild‐type protein.
**Figure S11:** Survey of residual differences between native *Mj*TyrRS, AzAlaRS, 5*O*MeW_RS, and 5CNW_RS enzymes (for chemical structures of related cognate ncAAs, see Figure 1 in the manuscript.).
**Figure S12:** Simple structural models of monomeric *Methanocaldococcus jannaschii* (*Mj*TyrRS; PDB code: 1J1U; sky blue with yellow ligand) and its selected active site mutations. In this way, a graphical visualization of the sequence data from Figure S11 between native, AzAlaRS, 5*O*MeW_RS, and 5CNW_RS enzymes is used to highlight the difference between the active sites of these enzymes. The active site of Azulenylalanyl‐tRNA synthetase (AzAlaRS; PDB code: 5NSF) with residues in split pea green and ligand (Azulenylalanine, AzAla) in bright orange. The hypothetical model of the active site of 5*O*MeW_RS (created in Pymol, based on 5NSF structure, without calculation) with residues highlighted in violet purple and 5*O*MeW ligand in pale yellow (created in ChemDraw, with dihedrals adjusted to best match the AzAla ligand in AzAlaRS structure). Finally, the hypothetical model of the active site of the enzyme 5CNW_RS (dark teal with yellow‐orange ligand), created in the same way as 5*O*MeW_RS above.
