# Supplementary material for: Orthogonal translation with 5‐cyanotryptophan as an infrared probe for local structural information, electrostatics, and hydrogen bonding
Source: Protein Sci. 2023 Jul 1;32(7):e4705. doi: 10.1002/pro.4705 (PMC10288556; doi:10.1002/pro.4705)
Supplement: Supplementary file 1 — Figure S1: Whole‐cell fluorescence assay with 5CNW_RS variants randomized at position 32. 96 single colonies were assayed in the presence 5CNW and sfGFP expression was monitored via fluorescence measurements. The bar on the far‐right side displays the fluorescence yielded with 5CNW_RS (WT). Figure S2: Whole‐cell fluorescence assay with 5CNW_RS variants randomized at position 65. 96 single colonies were assayed in the presence 5CNW and sfGFP expression was monitored via fluorescence measurements. The bar on the far‐right side displays the fluorescence yielded with 5CNW_RS (WT). Figure S3: Whole‐cell fluorescence assay with 5CNW_RS variants randomized at position 70. 96 Single colonies were assayed in the presence 5CNW and sfGFP expression was monitored via fluorescence measurements. The bar on the far‐right side displays the fluorescence yielded with 5CNW_RS (WT). Figure S4: Whole‐cell fluorescence assay with 5CNW_RS variants randomized at position 108. 96 single colonies were assayed in the presence 5CNW and sfGFP expression was monitored via fluorescence measurements. The bar on the far‐right side displays the fluorescence yielded with 5CNW_RS (WT). Figure S5: Whole‐cell fluorescence assay with 5CNW_RS variants randomized at position 109. 96 single colonies were assayed in the presence 5CNW and sfGFP expression was monitored via fluorescence measurements. The bar on the far‐right side displays the fluorescence yielded with 5CNW_RS (WT). Figure S6: Whole‐cell fluorescence assay with 5CNW_RS variants randomized at position 162. 96 single colonies were assayed in the presence 5CNW and sfGFP expression was monitored via fluorescence measurements. The bar on the far‐right side displays the fluorescence yielded with 5CNW_RS (WT). Figure S7: Whole‐cell fluorescence assay with 5CNW_RS variants randomized at position 167. 96 single colonies were assayed in the presence 5CNW and sfGFP expression was monitored via fluorescence measurements. The bar on the far‐right side displ [file PRO-32-e4705-s001.docx]

***Supplementary Information***

**Orthogonal translation with 5-cyanotryptophan as an infrared probe for local structural information, electrostatics, and hydrogen bonding**

**Freiherr von Sass, Georg Johannes^1^; Blain-Hartung, Matthew^2^; Baumann, Tobias^1^;**

**Forest, Katrina^3^; Hildebrandt, Peter^2^; Budisa, Nediljko^1,4^***

^1^Technische Universität Berlin, Institut für Chemie/Biokatalyse, Sekr. CL1, Müller-Breslau-Str. 10, 10623, Berlin, Germany.

^2^Technische Universität Berlin, Institut für Chemie / Physikalische Chemie, Sekr. PC 14, Str. des 17. Juni 135, 10623, Berlin, Germany.

^3^University of Wisconsin-Madison, Department of Bacteriology, 1550 Linden Dr., Madison WI 53706, USA.

^4^Department of Chemistry, University of Manitoba, 144 Dysart Rd, 360 Parker Building, R3T 2N2 Winnipeg, Manitoba, Canada.

Nucleotide sequences

5*O*MeW_RS

ATGGATGAATTTGAAATGATTAAACGCAACACCAGCGAAATCATTAGCGAAGAAGAACTGCGCGAAGTCCTGAAAAAAGATGAAAAAAGCGCCGGTATTGGCTTTGAACCGAGCGGTAAAATTCATCTGGGTCATTATCTGCAAATCAAAAAAATGATTGATCTGCAAAACGCAGGCTTTGATATTATCATTTGGCTGGGCGATCTGGGCGCCTATCTGAATCAGAAAGGCGAACTGGATGAAATTCGCAAAATTGGCGACTATAACAAAAAAGTGTTTGAGGCAATGGGCCTGAAAGCCAAATATGTGTATGGAAGCGAAGAGAGTCTGGATAAAGATTATACCCTGAACGTGTATCGTCTGGCACTGAAAACCACCCTGAAACGTGCACGTCGTAGCATGGAACTGATTGCACGTGAAGATGAAAATCCGAAAGTTGCCGAAGTGATCTATCCGATTATGCAGGTGAACGCCATCCATTATGATGGCGTCGATGTTGCCGTGGGTGGTATGGAACAGCGCAAAATCCATATGCTGGCACGTGAACTGCTGCCGAAAAAAGTCGTGTGCATTCATAATCCGGTTCTGACCGGTCTGGATGGTGAAGGCAAAATGAGCAGCAGCAAAGGTAACTTTATTGCCGTTGATGATAGTCCGGAAGAAATTCGTGCCAAAATCAAAAAAGCATATTGTCCGGCAGGCGTTGTTGAAGGTAATCCGATTATGGAAATCGCCAAATACTTTCTGGAATATCCGCTGACCATTAAACGTCCGGAAAAATTTGGTGGTGATCTGACCGTTAATAGCTATGAAGAACTGGAAAGCCTGTTTAAAAACAAAGAACTGCATCCGATGCGTCTGAAAAATGCAGTTGCAGAAGAACTGATCAAAATCCTGGAACCGATTCGTAAACGTCTGTAA

5CNW_RS

ATGGATGAATTTGAAATGATTAAACGCAACACCAGCGAAATCATTAGCGAAGAAGAACTGCGCGAAGTCCTGAAAAAAGATGAAAAAAGCGCCGGTATTGGCTTTGAACCGAGCGGTAAAATTCATCTGGGTCATTATCTGCAAATCAAAAAAATGATTGATCTGCAAAACGCAGGCTTTGATATTATCATTTGGCTGGGCGATCTGGGCGCCTATCTGAATCAGAAAGGCGAACTGGATGAAATTCGCAAAATTGGCGACTATAACAAAAAAGTGTTTGAGGCAATGGGCCTGAAAGCCAAATATGTGTATGGAAGCGAATGGTTTCTGGATAAAGATTATACCCTGAACGTGTATCGTCTGGCACTGAAAACCACCCTGAAACGTGCACGTCGTAGCATGGAACTGATTGCACGTGAAGATGAAAATCCGAAAGTTGCCGAAGTGATCTATCCGATTATGCAGGTGAACGCCATCCATTATGATGGCGTCGATGTTGCCGTGGGTGGTATGGAACAGCGCAAAATCCATATGCTGGCACGTGAACTGCTGCCGAAAAAAGTCGTGTGCATTCATAATCCGGTTCTGACCGGTCTGGATGGTGAAGGCAAAATGAGCAGCAGCAAAGGTAACTTTATTGCCGTTGATGATAGTCCGGAAGAAATTCGTGCCAAAATCAAAAAAGCATATTGTCCGGCAGGCGTTGTTGAAGGTAATCCGATTATGGAAATCGCCAAATACTTTCTGGAATATCCGCTGACCATTAAACGTCCGGAAAAATTTGGTGGTGATCTGACCGTTAATAGCTATGAAGAACTGGAAAGCCTGTTTAAAAACAAAGAACTGCATCCGATGCGTCTGAAAAATGCAGTTGCAGAAGAACTGATCAAAATCCTGGAACCGATTCGTAAACGTCTGTAA

**Figure S1**: Whole-cell fluorescence assay with 5CNW_RS variants randomized at position 32. 96 single colonies were assayed in the presence 5CNW and sfGFP expression was monitored via fluorescence measurements. The bar on the far-right side displays the fluorescence yielded with 5CNW_RS (WT).

**Figure S2**: Whole-cell fluorescence assay with 5CNW_RS variants randomized at position 65. 96 single colonies were assayed in the presence 5CNW and sfGFP expression was monitored via fluorescence measurements. The bar on the far-right side displays the fluorescence yielded with 5CNW_RS (WT).

**Figure S3**: Whole-cell fluorescence assay with 5CNW_RS variants randomized at position 70. 96 Single colonies were assayed in the presence 5CNW and sfGFP expression was monitored via fluorescence measurements. The bar on the far-right side displays the fluorescence yielded with 5CNW_RS (WT).

**Figure S4**: Whole-cell fluorescence assay with 5CNW_RS variants randomized at position 108. 96 single colonies were assayed in the presence 5CNW and sfGFP expression was monitored via fluorescence measurements. The bar on the far-right side displays the fluorescence yielded with 5CNW_RS (WT).

**Figure S5**: Whole-cell fluorescence assay with 5CNW_RS variants randomized at position 109. 96 single colonies were assayed in the presence 5CNW and sfGFP expression was monitored via fluorescence measurements. The bar on the far-right side displays the fluorescence yielded with 5CNW_RS (WT).

**Figure S6:** Whole-cell fluorescence assay with 5CNW_RS variants randomized at position 162. 96 single colonies were assayed in the presence 5CNW and sfGFP expression was monitored via fluorescence measurements. The bar on the far-right side displays the fluorescence yielded with 5CNW_RS (WT).

**Figure S7**: Whole-cell fluorescence assay with 5CNW_RS variants randomized at position 167. 96 single colonies were assayed in the presence 5CNW and sfGFP expression was monitored via fluorescence measurements. The bar on the far-right side displays the fluorescence yielded with 5CNW_RS (WT).

**Figure S8**: Whole-cell fluorescence assay with 5CNW_RS variants randomized at position 180. 96 single colonies were assayed in the presence 5CNW and sfGFP expression was monitored via fluorescence measurements. The bar on the far-right side displays the fluorescence yielded with 5CNW_RS (WT).

Whole-cell fluorescence assay with selected 5CNW_RS variants

**Figure S9:** Whole-cell fluorescence assay with selected 5CNW_RS variants tested for ribosomal incorporation of 5CNW. The assay was performed with supplemented ncAA (+5CNW) or without its addition (-5CNW). It took place in biological triplicates and the standard deviation is depicted as error bars. The fluorescence obtained by expression of the initial 5OMeW_RS (WT) enzyme in presence of 5CNW was normalized to 100 %.

HPLC-ESI-QTOF-MS of sfGFP with incorporated 5CNW

**Figure S10:** HPLC-ESI-QTOF-MS analysis of 5CNW incorporation into sfGFP. Deconvoluted mass of purified SUMO-sfGFP WT and SUMO-sfGFP proteins with 5CNW incorporated at position 2. The measured and expected molecular masses are as follows: SUMO-sfGFP WT: measured: 40208 Da, expected: 40208.15 Da. Sumo-sfGFP_R2(5CNW): measured: 40263 Da, expected: 40263.17 Da. The secondary peaks (40229 Da and 40284 Da, respectively) represent sfGFP derivatives with immature chromophore, as evident from the wild-type protein.

**
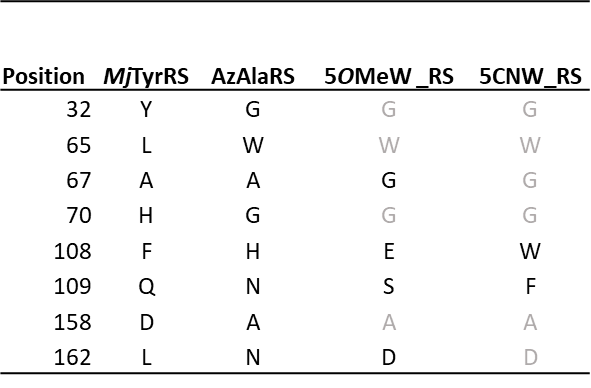
**

**Figure S11:** Survey of residual differences between native *Mj*TyrRS, AzAlaRS, 5*O*MeW_RS, and 5CNW_RS enzymes (for chemical structures of related cognate ncAAs, see Fig. 1 in the manuscript.).


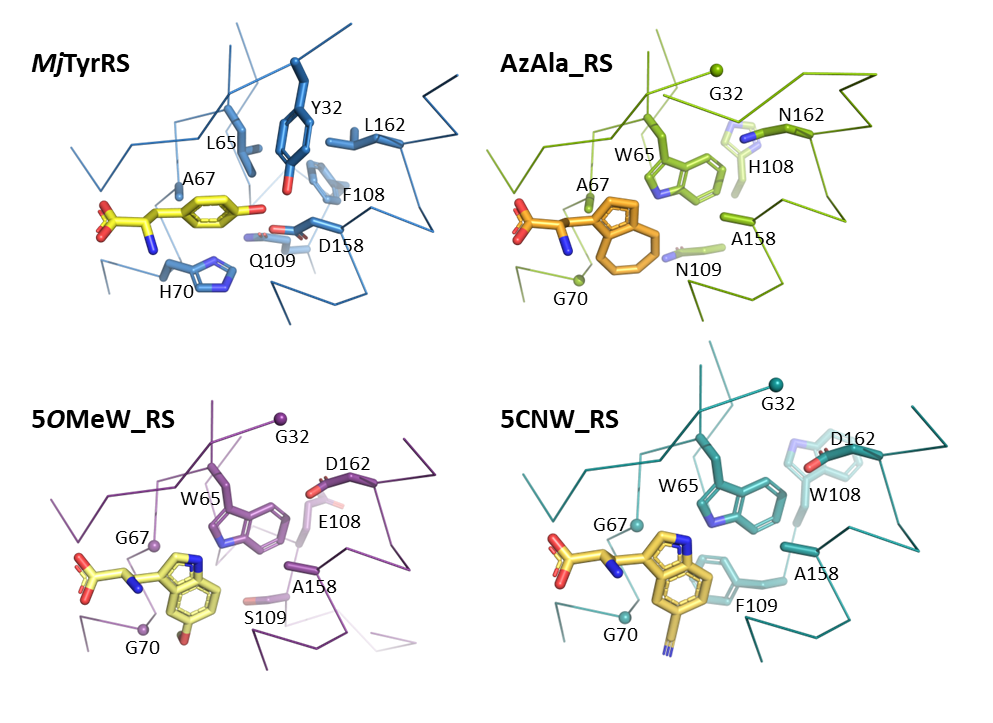


**Figure S12:** Simple structural models of monomeric *Methanocaldococcus jannaschii* (*Mj*TyrRS; PDB code: 1J1U; sky blue with yellow ligand) and its selected active site mutations. In this way, a graphical visualization of the sequence data from Fig. S11 between native, AzAlaRS, 5*O*MeW_RS, and 5CNW_RS enzymes is used to highlight the difference between the active sites of these enzymes. The active site of Azulenylalanyl-tRNA synthetase (AzAlaRS; PDB code: 5NSF) with residues in split pea green and ligand (Azulenylalanine, AzAla) in bright orange. The hypothetical model of the active site of 5*O*MeW_RS (created in Pymol, based on 5NSF structure, without calculation) with residues highlighted in violet purple and 5*O*MeW ligand in pale yellow (created in ChemDraw, with dihedrals adjusted to best match the AzAla ligand in AzAlaRS structure). Finally, the hypothetical model of the active site of the enzyme 5CNW_RS (dark teal with yellow-orange ligand), created in the same way as 5*O*MeW_RS above.

OLIGONUCLEOTIDES

| Name | Sequence (5’ to 3’) |
| --- | --- |
| MjTyrRS-NotI_fw | AGGAGCGGCCGCATGGATGAATTTGAAATGATTAAACGC |
| MjTyrRS-NotI_rv | AGGAGCGGCCGCTTACAGACGTTTACGAATCGGTTC |
| MjTyrRS_E32NNK-BsaI_fw | TATGGTCTCTCGCCNNKATTGGCTTTGAACCGAGCG |
| MjTyrRS_E32NNK-BsaI_rv | TATGGTCTCTGGCGCTTTTTTCATCTTTTTTCAGGAC |
| MjTyrRS_W65NNK-BsaI_fw | TATGGTCTCTCATTNNKCTGGGCGATCTGGGCGC |
| MjTyrRS_W65NNK-BsaI_rv | TATGGTCTCTAATGATAATATCAAAGCCTGCGTTTTGC |
| MjTyrRS_G70NNK-BsaI_fw | TATGGTCTCTTCTGNNKGCCTATCTGAATCAGAAAGGCGAAC |
| MjTyrRS_G70NNK-BsaI_rv | TATGGTCTCTCAGATCGCCCAGCCAAATGATAATATCAAAG |
| MjTyrRS_S109NNK-BsaI_fw | TATGGTCTCTAGAGNNKCTGGATAAAGATTATACCCTGAACGTGTATC |
| MjTyrRS_S109NNK-BsaI_rv | TATGGTCTCTCTCTTCGCTTCCATACACATATTTGGCTTTC |
| MjTyrRS_E108NNK-BsaI_fw | TATGGTCTCTCGAANNKAGTCTGGATAAAGATTATACCCTGAACGTG |
| MjTyrRS_E108NNK-BsaI_rv | TATGGTCTCTTTCGCTTCCATACACATATTTGGCTTTCAGG |
| MjTyrRS_D162NNK-BsaI_fw | TATGGTCTCTTTATNNKGGCGTCGATGTTGCCGTG |
| MjTyrRS_D162NNK-BsaI_rv | TATGGTCTCTATAATGGATGGCGTTCACCTGCATAATCG |
| MjTyrRS_A167NNK-BsaI_fw | TATGGTCTCTTGTTNNKGTGGGTGGTATGGAACAGC |
| MjTyrRS_A167NNK-BsaI_rv | TATGGTCTCTAACATCGACGCCATCATAATGG |
| MjTyrRS_A180NNK-BsaI_fw | TATGGTCTCTGCTGNNKCGTGAACTGCTGCCGAAAAAAGTC |
| MjTyrRS_A180NNK-BsaI_rv | TATGGTCTCTCAGCATATGGATTTTGCGCTGTTCC |
| 5-CN-TrpRS_E108W-S109A_fw | CGAATGGGCGCTGGATAAAGATTATACCCTGAACGTG |
| 5-CN-TrpRS_E108W-S109A_rv | CAGCGCCCATTCGCTTCCATACACATATTTGG |
| 5-CN-TrpRS_E108W-S109F_fw | CGAATGGTTTCTGGATAAAGATTATACCCTGAACGTG |
| 5-CN-TrpRS_E108W-S109F_rv | CAGAAACCATTCGCTTCCATACACATATTTGG |
| 5-CN-TrpRS_E108W-S109G_fw | CGAATGGGGCCTGGATAAAGATTATACCCTGAACGTG |
| 5-CN-TrpRS_E108W-S109G_rv | CAGGCCCCATTCGCTTCCATACACATATTTGG |
| 5-CN-TrpRS_E108W-S109L_fw | CGAATGGCTGCTGGATAAAGATTATACCCTGAACGTG |
| 5-CN-TrpRS_E108W-S109L_rv | CAGCAGCCATTCGCTTCCATACACATATTTGG |

Non-canonical amino acids used to test the substrate tolerance of CNW_RS

| **Non-canonical amino acid** | **CAS number** | **Vendor** |
| --- | --- | --- |
| 5-cyano-L-tryptophan (5CNW) | 139393-02-5 | Amatek Chemicals (Jiangsu, China) |
| 5-Cl-DL-tryptophan | 154-07-4 | Biosynth Carbosynth (Berkshire, UK) |
| 5-Br-DL-tryptophan | 6548-09-0 | Biosynth Carbosynth (Berkshire, UK) |
| 5-F-DL-tryptophan | 154-08-5 | Sigma-Aldrich (Taufkirchen, Germany) |
| 5-OMe-tryptophan | 28052-84-8 | Biosynth Carbosynth (Berkshire, UK) |
| 5-methyl-DL-tryptophan | 951-55-3 | Biosynth Carbosynth (Berkshire, UK) |
| 5-amino-tryptophan | 6383-69-3 | Biosynth Carbosynth (Berkshire, UK) |
| 5-nitro-tryptophan | 6525-46-8 | Biosynth Carbosynth (Berkshire, UK) |
| 5-OH-tryptophan | 145224-90-4 | Biosynth Carbosynth (Berkshire, UK) |
| 6-Cl-DL-tryptophan | 56632-86-1 | Biosynth Carbosynth (Berkshire, UK) |
| 1-methyl-tryptophan | 26988-72-7 | Biosynth Carbosynth (Berkshire, UK) |
| 7-Br-tryptophan | 75816-19-2 | Amatek Chemicals (Jiangsu, China) |
| 4-amino-phenylalanine | 2922-41-0 | Sigma-Aldrich (Taufkirchen, Germany) |
| 4-I-DL-phenylalanine | 24250-85-9 | Bachem (Bubendorf, Switzerland) |
| 4-azido-phenylalanine | 33173-53-4 | Bachem (Bubendorf, Switzerland) |
| 4-nitro-phenylalanine | 949-99-5 | Bachem (Bubendorf, Switzerland) |
| 3-Cl-tyrosine | 7423-93-0 | Sigma-Aldrich (Taufkirchen, Germany) |
| 3-Br-tyrosine | 54788-30-6 | abcr GmbH (Karlsruhe, Germany) |
| 3,5-Cl-tyrosine | 15106-62-4 | TCI chemicals (Zwijndrecht, Belgium) |
| O-tert-butyl-tyrosine | 18822-59-8 | TCI chemicals (Zwijndrecht, Belgium) |
| 3,5-I-tyrosine | 18835-59-1 | TCI chemicals (Zwijndrecht, Belgium) |
| 3-nitro-tyrosine | 621-44-3 | Carl Roth (Karslruhe, Germany) |
| O-benzyl-tyrosine | 16652-64-5 | Bachem (Bubendorf, Switzerland) |
| 3-amino-tyrosine | 23279-22-3 | Sigma-Aldrich (Taufkirchen, Germany) |
| O-methyl-tyrosine | 6230-11-1 | Sigma-Aldrich (Taufkirchen, Germany) |
| S-benzyl-cysteine | 3054-01-1 | VWR Int.GmbH (Darmstadt, Germany) |
